# Intravesical Instillation of Azacitidine Suppresses Tumor Formation through TNF-R1 and TRAIL-R2 Signaling in Genotoxic Carcinogen-Induced Bladder Cancer

**DOI:** 10.3390/cancers13163933

**Published:** 2021-08-04

**Authors:** Shao-Chuan Wang, Ya-Chuan Chang, Min-You Wu, Chia-Ying Yu, Sung-Lang Chen, Wen-Wei Sung

**Affiliations:** 1 Department of Urology, Chung Shan Medical University Hospital, Taichung 40201, Taiwan; cshy764@csh.org.tw (S.-C.W.); cshy650@csh.org.tw (S.-L.C.); 2 School of Medicine, Chung Shan Medical University, Taichung 40201, Taiwan; raptor7037@gmail.com (Y.-C.C.); s0701130@gm.csmu.edu.tw (M.-Y.W.); cyyu2015@gmail.com (C.-Y.Y.); 3 Institute of Medicine, Chung Shan Medical University, Taichung 40201, Taiwan

**Keywords:** bladder cancer, azacitidine, intravesical instillation, TNF-R1, TRAIL-R2

## Abstract

**Simple Summary:**

Approximately 70% of all bladder cancer is diagnosed as non-muscle invasive bladder cancer and can be treated by transurethral resection of the bladder tumor, followed by intravesical instillation chemotherapy. Bacille Calmette-Guérin (BCG) is the first-line agent for intravesical instillation, but its accessibility has been limited for years due to a BCG shortage. Here, our aim was to evaluate the therapeutic role of intravesical instillation of azacitidine, a DNA methyltransferase inhibitor, in bladder cancer. Cell model experiments showed that azacitidine inhibited TNFR1 downstream pathways to downregulate HIF-1α, claspin, and survivin. Concomitant upregulation of the TRAIL R2 pathway by azacitidine ultimately drove the tumor cells to apoptosis. Rats with genotoxic carcinogen-induced bladder cancer showed a significantly reduced in vivo tumor burden after intravesical instillation of azacitidine. These findings might support further clinical trials of azacitidine in bladder cancer.

**Abstract:**

Azacitidine, an inhibitor of DNA methylation, shows therapeutic effects against several malignancies by inducing apoptosis and inhibiting tumor cell proliferation. However, the anti-tumor effects of azacitidine on urinary bladder urothelial carcinoma (UBUC), especially following intravesical instillation (IVI), are not established. Here, UBUC cell lines were used to analyze the in vitro therapeutic effects of azacitidine. Potential signaling pathways were investigated by antibody arrays and Western blotting. The N-butyl-N-(4-hydroxybutyl) nitrosamine (BBN)-induced rat UBUC model was used for in vivo quantitative analysis of tumor burden. Azacitidine significantly inhibited DNMT expression in UBUC cell lines and reduced cell viability and clonogenic activity, as determined by MTT and colony formation assays, while also inducing significant cytotoxic effects in the form of increased sub-G1 and Annexin V-PI populations (all *p* < 0.05). Antibody arrays confirmed the in vitro suppression of TNF-R1 and the induction of TRAIL-R2 and their downstream signaling molecules. TNF-R1 suppression reduced claspin and survivin expression, while TRAIL-R2 activation induced cytochrome C and caspase 3 expression. Rats with BBN-induced bladder cancer had a significantly reduced tumor burden and Ki67 index following IVI of azacitidine (*p* < 0.01). Our study provides evidence for a reduction in BBN-induced bladder cancer by IVI of azacitidine through alterations in the TRAIL-R2 and TNF-R1 signaling pathways. These findings might provide new insights for further clinical trials.

## 1. Introduction

Urinary bladder urothelial carcinoma (UBUC), also known as bladder cancer, is a highly prevalent malignancy worldwide. In 2020, the estimated new cases and deaths were 81,400 and 17,980, respectively, in the United States [1]. Non-muscle invasive bladder cancer (NMIBC) accounts for 70% of all bladder cancer diagnoses [2], and the management of NMIBC consists mostly of tumor excision via transurethral resection of the bladder tumor (TURBT). Subsequent intravesical instillation (IVI) of chemotherapeutic agents is performed within 24 h after surgery to eliminate residual cancer cells [3].

IVI therapy directly instills chemotherapy or immunotherapy agents, such as mitomycin C and Bacille Calmette-Guérin (BCG), into the bladder [2]. BCG is the first-line treatment for NMIBC, and it significantly reduces the recurrence of malignancy [4]. BCG has several disadvantages, such as side effects that include a localized inflammatory response and BCG infection; however, a shortage of BCG agent currently limits accessibility to this treatment [5,6]. In addition, the tumor recurrence rate remains at about 40% following chemotherapy instillation [7], indicating that any reduction in the rate of UBUC recurrence will require the exploration of alternative agents or agent combinations for IVI bladder cancer therapy.

The progression of many cancers involves epigenetic abnormalities, such as aberrant DNA or histone methylation, which result in disturbances of gene expression [8,9]. Epigenetic therapy, therefore, plays an essential role in cancer treatment, and the vast majority of epigenetic drugs are histone deacetylase inhibitors (HDACi) and DNA methyltransferase inhibitors (DNMTi) [10]. Ongoing or completed clinical trials of epigenetic drugs for treating bladder malignancy have mainly involved HDACi treatments, with only about 20% investigating DNMTi efficacy [11]. Similarly, very few studies have explored the intravesical instillation of DNMTi as adjuvant therapy.

One promising DNMTi inhibitor is azacitidine, a type of hypomethylating agent that incorporates into DNA and RNA. It also binds to DNMT1 as an irreversible inhibitor [12], thereby preventing DNA methylation and reactivating tumor suppressor genes [13]. Azacitidine has been used as therapy for myelodysplastic syndromes and it improves outcomes for elderly patients diagnosed with acute myeloid leukemia [14,15]. Azacitidine also has therapeutic effects on solid tumors [16]. Currently, most clinical trials have focused on azacitidine as therapy for leukemia, but a few studies have examined the potential of intravenous injection as therapy for urologic malignancies, such as castration-resistant prostate cancer [17].

Azacitidine promotes the expression of tumor suppressor genes and inhibits the proliferation of bladder cancer cell lines, suggesting a potential therapeutic role in UBUC [18]. Azacitidine can also increase the sensitivity of drug-resistant bladder urothelial carcinoma cells to chemotherapy agents [19]. We hypothesized that azacitidine might show an anti-tumor effect and reduce the tumor recurrence rate in UBUC. The aim of the present study was to examine the anti-tumor effects of azacitidine on UBUC cell lines and to conduct in vivo experiments to assess the feasibility of using IVI of azacitidine as a treatment for UBUC.

## 2. Materials and Methods

### 2.1. Cell Culture

Two UBUC cell lines, T24 and UMUC3, were purchased from The American Type Culture Collection (Manassas, VA, USA) and stored according to the supplier’s instructions. Cells were grown in RPMI medium containing 10% fetal bovine serum, 100 U/mL penicillin, 100 μg/mL streptomycin, 2 g/mL NaHCO_3,_ 1 mM sodium pyruvate, and 0.1 mM non-essential amino acids. All cell lines were cultured at 37 °C and supplied with 5% CO_2_.

### 2.2. MTT Assays

The MTT assay was performed to detect the cytotoxicity of azacitidine. Briefly, 2 × 10^4^ cells were seeded into 96-well plates, incubated overnight, and exposed to azacitidine (0, 1, and 10 μM) for 1 or 24 h and harvested on the 72nd hour. MTT solution (0.5 mg/mL) was added to the wells and incubated for 3 h at 37 °C. The reaction was ended by the removal of the supernatant, followed by dissolving the formazan product in DMSO. Optical density was measured with an ELISA reader using a 570 nm filter. Each experiment was performed in triplicate and the cell viability of each group was calculated and analyzed compared to the control.

### 2.3. Clonogenic Assay

T24 (250 cells) and UMUC3 (500 cells) treated with azacitidine (0, 1, and 10 μM) were seeded into each well of a 6-well plate and incubated for 3 or 10 days. The resulting colonies were fixed with 95% ethanol for 20 min and stained with 20% Giemsa solution for 30 min at room temperature, as previously described [20]. Each experiment was performed in triplicate and colony counts were calculated for each group.

### 2.4. Flow Cytometry Analysis

Flow cytometry was conducted with a FACSCanto™ II Cell Analyzer (BD Biosciences, Franklin Lakes, NJ, USA) to determine the cell cycle distribution and percentage of apoptotic cells in each group, as previously described [21]. T24 and UMUC3 cells were seeded in 6-well plates and treated with azacitidine (0, 1, and 10 μM) for 72 h. For cell cycle distribution analysis, cells were fixed in pre-chilled 70% ethanol. The fixed cells were then re-suspended in PBS containing 0.4 µg/mL PI and 0.5 mg/mL RNase, and analyzed by flow cytometry. Apoptosis was analyzed using an Annexin V-FITC apoptosis detection kit (Strong Biotech Corporation, Taipei, Taiwan) according to the recommended protocol. Cells were re-suspended in binding buffer (100 μL), and 2 μL Annexin V-FITC and 2 μL PI were added. After a 15 min reaction in the dark, the cell apoptosis rate was evaluated by flow cytometry. Each experiment was performed in triplicate. The percentage of DNA content at different phases of the cell cycle and the cell apoptosis rate were analyzed using FlowJo Software (BD Biosciences, Franklin Lakes, NJ, USA).

### 2.5. Hoechst Staining Assay

Hoechst 33,342 staining was conducted to detect cell apoptosis by cell morphology observation. The UBUC cell lines were seeded into 6-well plates at a density of 2.5 × 10^5^ cells/well and incubated overnight. The cells were then treated with azacitidine (0, 1, and 10 μM) for 24 h and then harvested at 48 h. The cells were thoroughly washed with phosphate buffered saline and stained with Hoechst 33,342 (10 μg/mL, Invitrogen), followed by incubation for 20 min at 37 °C. Images were captured with a fluorescence microscope (ImageXpress PICO, Molecular Devices, LLC, San Jose, CA, USA, excitation wavelength of 350–390 nm, emission wavelength of 420–480 nm) at 20× magnification. Five different fields were selected, and the condensed nuclei in each field were counted against the total number of nuclei in the field. The percentages of condensed nuclei were plotted for further analysis.

### 2.6. Immunoblotting Assays

T24 and UMUC3 cells used for immunoblotting were treated with azacitidine (0, 1, and 10 μM; 24 h) and harvested for further investigation. Proteins were extracted with RIPA lysis buffer containing complete protease inhibitor cocktail tablets (Roche Applied Science, Mannheim, Germany) dissolved in PBS. The cells were lysed by sonication on ice, the extract was centrifuged for 20 min at 13,800× *g* and 4 °C, and the supernatant was stored at −80°C. The protein concentration in the supernatant was measured with the Bio-Rad Protein Assay (Bio-Rad Laboratories Inc., Contra Costa, CA, USA). Equal amounts of protein (15 μg) were then loaded onto sodium dodecyl sulfate polyacrylamide gel electrophoresis (SDS-PAGE) gels, separated by electrophoresis, and then transferred from the gel to Immobilon^TM^-P Transfer Membranes (Merck Millipore, Burlington, MA, USA). The membranes were blocked with 5% non-fat milk and then incubated overnight at 4 °C with the relevant primary antibodies. The membranes were washed with Tris-buffered saline containing Tween 20 (TBST) and then incubated with horseradish-peroxidase-conjugated secondary antibody for 1 h. The membranes were then washed with TBST and reacted with Immobilon^TM^-Western Chemiluminescent HRP Substrate (Merck Millipore, Burlington, MA, USA). The results were detected with a GE Healthcare ImageQuant LAS4000 instrument, and the band densities were quantified with AlphaEaseFC software using β-actin for normalization of the results. All antibodies were purchased from ABclonal Science, Inc. (Woburn, MA, USA) and Taiclone Biotech (Tainan, Taiwan).

### 2.7. Human Apoptosis Array for Proteome Profiling

Proteome profiling was conducted using a Human Apoptosis Proteome Profiler™ array (R&D Systems, Minneapolis, MN, USA), consisting of a nitrocellulose membrane with duplicate spots of 35 apoptosis-related proteins. T24 and UMUC3 cells were treated with or without azacitidine (10 μM) for 24 h, lysed, and 400 μg total protein was used for each array following the manufacturer’s protocol. The membranes were incubated with horseradish-peroxidase-conjugated antibody, followed by a chemiluminescent detection reagent, and detected with the GE Healthcare ImageQuant LAS4000 instrument (Cytiva, Marlborough, MA, USA). Each experiment was repeated in duplicate, and the integrated density of the spots was quantitated using Image J software (National Institutes of Health, Bethesda, MD, USA).

### 2.8. BBN-Induced Bladder Cancer Animal Model (Azacitidine Treatment)

Female Sprague–Dawley rats weighing between 200 and 230 g were purchased from the National Laboratory Animal Center. Urinary bladder tumors were induced by administration of 0.05% N-butyl-N-(4-hydroxybutyl) nitrosamine (BBN, Sunshine Chemical), dissolved in drinking water, for 20 weeks, as described previously [22]. The therapeutic effects of intravesical azacitidine (CAS no.320-67-2, Sigma-Aldrich, St. Louis,, MO, USA) on bladder tumorigenicity in BBN-induced rats were determined by IVI of azacitidine at 1, 5, 9, 13, and 17 weeks. The IVI protocol was as follows: all rats were anesthetized by intraperitoneal administration of Rompun (6 mg/kg) and Zoletil (30 mg/kg). A sterile polyethylene catheter (PE-50) was then inserted into the bladder through the urethra, and all urine was aspirated. The rats in the BBN-induced group underwent IVI with 0.2 mL azacitidine (0, 10, or 50 μM; *n* = 5, *n* = 6, *n* = 6, respectively; control without BBN, *n* = 3) under 1 h of continuous sedation. The non-BBN-induced group of rats underwent IVI with 0.2 mL PBS under 1 h of continuous sedation. The rats were euthanized by asphyxiation with CO_2_ 20 weeks after BBN induction. Dissected rat bladders were photographed and used for further analysis.

### 2.9. Hematoxylin and Eosin Staining

Bladder tissues were embedded in paraffin wax, cut into 3 µm sections, placed on slides, and stained with hematoxylin/eosin (H&E) by routine histopathological methods. Briefly, after deparaffinization and rehydration, the tissue sections were immersed in hematoxylin for 10 min and washed with tap water for 10 min. The slides were then immersed in eosin for 30 s and dehydrated by immersion in a series of increasing concentrations of alcohol. The sections were then immersed in xylene twice for 10 min each and coverslipped with mounting medium. The stained specimens were photographed using a TissueFAX Plus system (TissueGnostics, Vienna, Austria) and then analyzed histopathologically.

### 2.10. Immunohistochemistry

Bladder tissues were embedded in paraffin wax and cut into 3 µm sections. After deparaffinization and rehydration, antigen retrieval was performed by immersing the sections in citrate buffer (pH 6.0) for 30 min in an autoclave sterilizer and then cooling at room temperature for 10 min. Sections were incubated in 3% H_2_O_2_ for 10 min, followed by blocking with 10% goat serum for 1 h. The blocking buffer was removed, and the sections were incubated with the primary antibody at a 1:50 dilution (Anti-ki67 antibody #ab15580, Abcam, Cambridge, England) overnight at 4 °C. Subsequently, the sections were incubated with a biotinylated goat anti-rabbit IgG secondary antibody at a 1:100 dilution, followed by incubation with diaminobenzidine solution (#ab64238, Abcam) for 1 min. The sections were counterstained with hematoxylin (#ab220365, Abcam) for 20 s, dehydrated in a series of increasing concentrations of alcohol, immersed in xylene twice for 10 min each, and coverslipped with mounting medium. The stained specimens were photographed with a TissueFAX Plus system. The Ki-67 index was determined by expressing the numbers of positively stained cells in the bladder transitional epithelium as positive cell number/mm^2^, followed by analysis using TissueFAX Plus software.

### 2.11. Statistical Analysis

Statistical analysis was conducted using IBM SPSS software (version 20.0). Data were presented as the mean ± s.d. Student’s *t*-test was used for continuous or discrete data analysis, as described [20,23]. All statistical tests were two-sided (SEM), and values of *p* < 0.05 were considered statistically significant (*p < 0.05; ** *p* < 0.01; *** *p* < 0.001).

## 3. Results

### 3.1. Azacitidine Treatment of the UBUC Cell Lines Suppresses DNMT Expression and Cell Growth

**Figure 1:**
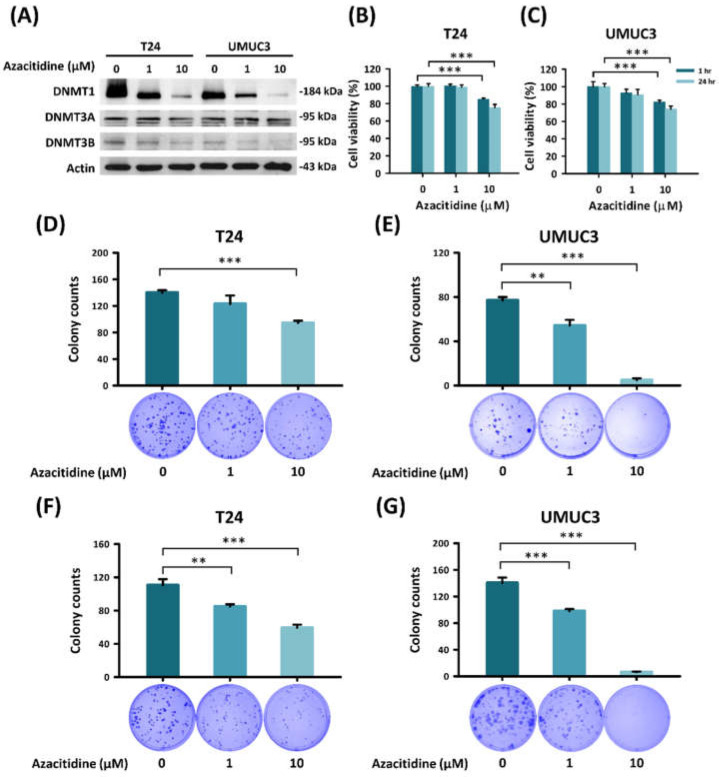
Azacitidine inhibits cell viability and clonogenic activity of UBUC cell lines. (**A**) Azacitidine inhibited DNMT1 expression. Expression of DNMT family proteins in UBUC cells treated with azacitidine (0, 1, and 10 µM) was examined by immunoblotting. (**B**,**C**) Azacitidine reduced cell viability of T24 and UMUC3 cells, as determined by the MTT assay. (**D**–**G**) Azacitidine inhibited colony formation by T24 and UMUC3 cells. Colony formation assays were conducted to measure cell growth changes in T24 and UMUC3 cells treated with azacitidine for 3 days (**D**,**E**) or 10 days (**F**,**G**) and harvested on the 10th day. Data for colony counts are presented in the upper panel. Data are shown as mean ± s.d (** *p* < 0.01; *** *p* < 0.001).

Azacitidine is known to suppress DNMT1 in several malignancies; therefore, we confirmed DNMT suppression by azacitidine in UBUC cells by determining the DNMT protein level by immunoblotting. As shown in Figure 1A, the expression of DNMT1 and DNMT3B in UBUC cells was decreased by treatment with 1 or 10 μM azacitidine.

The growth of azacitidine-treated UBUC cells was analyzed to test the cytotoxicity of azacitidine (Figure 1B,C). The MTT assay revealed that the viability of both T24 and UMUC3 cell types decreased after treatment with azacitidine for 1 h (T24: control vs. 10 μM, *p* = 0.001; UMUC3: control vs. 10 μM, *p* = 0.002) or 24 h (T24: control vs. 10 μM, *p* = 0.001; UMUC3: control vs. 10 μM, *p* = 0.001). Colony formation assays performed to analyze the clonogenic activity of UBUC cells (Figure 1D–G) revealed a decrease in colony formation (upper panel) by treatment with azacitidine for 3 days (T24: control vs. 1 and 10 μM, *p* = 0.91 and 0.001; UMUC3: control vs. 1 and 10 μM, *p* = 0.005 and 0.001) and 10 days (T24: control vs. 1 and 10 μM, *p* = 0.004 and 0.001; UMUC3: control vs. 1 and 10 μM, *p* = 0.001 and 0.001). The decreases in cell viability and clonogenic activity in response to azacitidine were dose dependent.

### 3.2. Azacitidine Induces Apoptosis of UBUC Cell Lines through Claspin and Survivin

**Figure 2:**
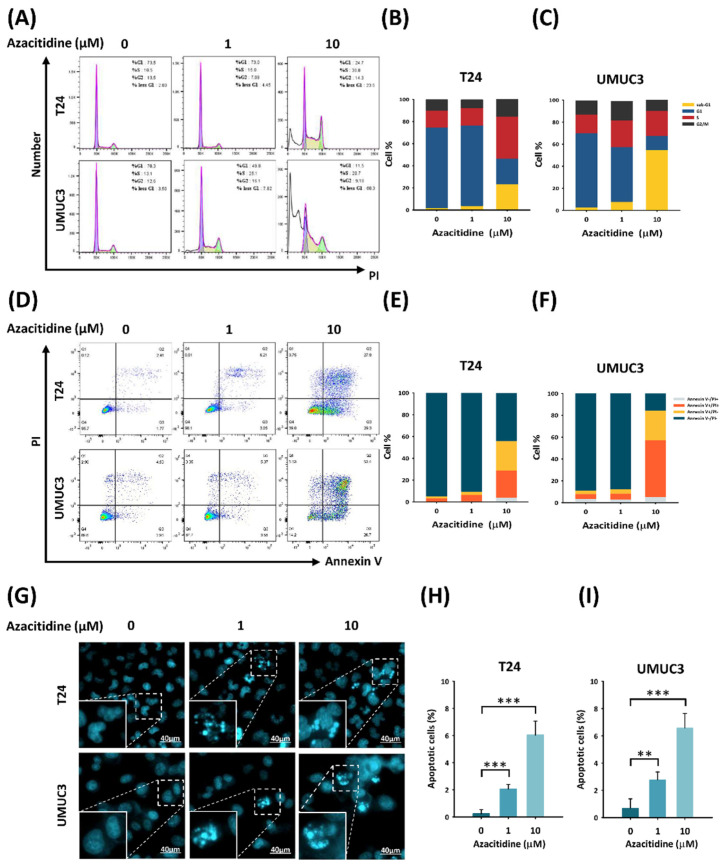
Azacitidine promotes apoptosis of UBUC cell lines. (**A**) Azacitidine increased the accumulation of UBUC cells in the sub-G1 phase. Cell cycle distributions, analyzed by flow cytometry, are shown for T24 (**B**) and UMUC3 (**C**) cells. (**D**) Azacitidine drove UBUC cell populations to early and late apoptosis, as determined by flow cytometry following dual staining with annexin V and PI. The proportions of annexin V^+/−^ and PI^+/−^ T24 (**E**) and UMUC3 (**F**) cells are shown. (**G**) The numbers of apoptotic UBUC cells increased in response to azacitidine treatment. Cell morphology changes caused by azacitidine were investigated by Hoechst 33,342 staining and viewing at 20× magnification. (**H**,**I**) Columns represent apoptotic cell percentages for T24 and UMUC3 cells. Data are shown as mean ± s.d. (** *p* < 0.01; *** *p* < 0.001).

We investigated whether cell growth was inhibited by suppression of cell proliferation or by promotion of cell death by examining the mechanism of cell growth suppression by azacitidine in UBUC cells. As shown in Figure 2A–C, the UBUC cells treated with azacitidine showed sub-G1 accumulation, indicating cell death (T24: control vs. 1 and 10 μM, *p* = 0.0.45 and 0.002; UMUC3: control vs. 1 and 10 μM, *p* = 0.001 and 0.001).

**Figure 3:**
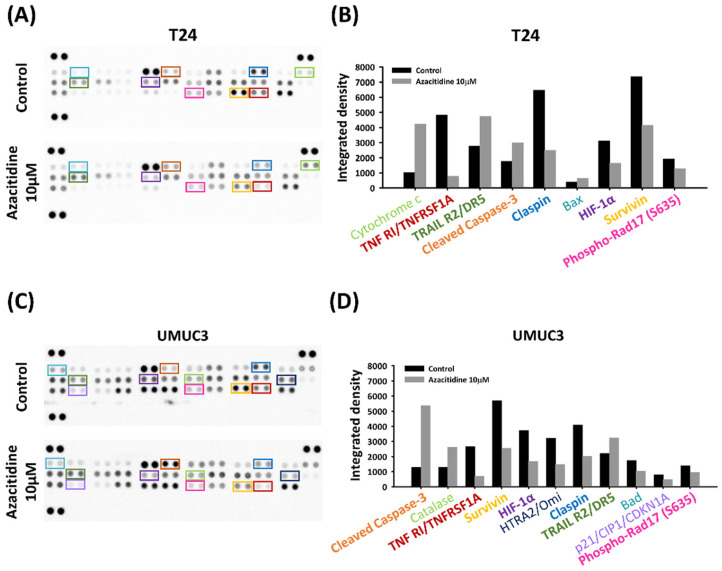
Apoptosis array determination of apoptosis-related protein changes due to azacitidine treatment. T24 (**A**) and UMUC3 (**C**) cells treated with azacitidine (0 and 10 μM) for 24 h were analyzed by apoptosis array. Quantitative analysis showed different changes in apoptotic markers in T24 (**B**) and UMUC3 (**D**) cells.

We distinguished between necrotic and apoptotic cells by dual staining with annexin V and PI and confirmed an apoptosis-inducing effect of azacitidine (Figure 2D–F). The numbers of early and late apoptotic cells increased with azacitidine treatment (T24: control vs. 1 and 10 μM, *p* = 0.024 and 0.001; UMUC3: control vs. 1 and 10 μM, *p* = 0.001 and 0.001). Visualization of apoptotic cells by Hoechst 33,342 staining and fluorescence microscopy (Figure 2G–I) revealed increased numbers of apoptotic cells following a 24 h azacitidine treatment (T24: control vs. 1 and 10 μM, *p* = 0.0001 and 0.0001; UMUC3: control vs. 1 and 10 μM, *p* = 0.002 and 0.0001). The apoptosis array (Figure 3A,C) identified tumor necrosis factor receptor 1 (TNF-R1) and TNF-related apoptosis-inducing ligand receptor 2 (TRAIL R2) as proteins whose expression was regulated by azacitidine in both UBUC cell lines. Claspin, survivin, and hypoxia-inducible factor 1-alpha (HIF-1α) expression was lower in cells treated with 10 μM azacitidine than in the control group (Figure 3B,D).

**Figure 4:**
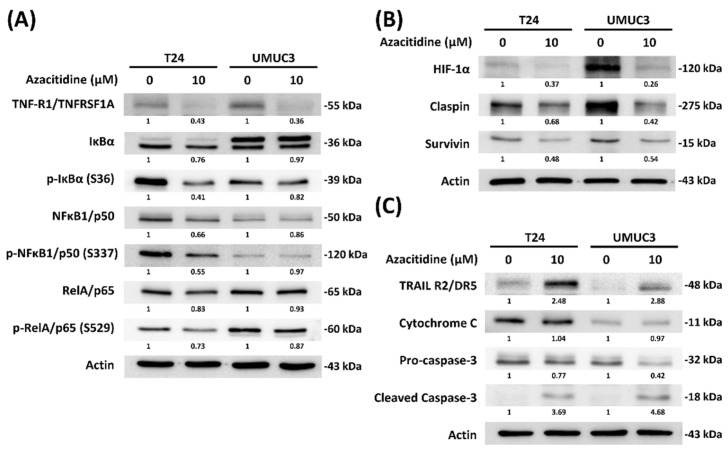
Azacitidine-facilitated apoptosis was associated with downregulation of the NF-κB pathway and upregulation of the TRAIL R2 pathway. (**A**) Azacitidine suppressed NF-κB-related protein expression in UBUC cells. (**B**) HIF-1α, claspin, and survivin expression were inhibited after azacitidine treatment. (**C**) TRAIL-related protein changes were evaluated after azacitidine treatment.

Investigation of the downstream pathway of TNF-R1 and TRAIL R2 by immunoblotting identified lower expression of a series of proteins involved in the NF-κB pathway (Figure 4A). The reduced expression of claspin, survivin, and HIF-1α confirmed their roles in the apoptosis pathway (Figure 4B). Evaluation of the expression of proteins in the TRAIL pathway revealed upregulation of the apoptosis markers cytochrome C and cleaved-caspase 3 following azacitidine treatment (Figure 4C).

### 3.3. Intravesical Instillation of Azacitidine Suppresses Bladder Cancer Growth in a BBN Rat Model

**Figure 5:**
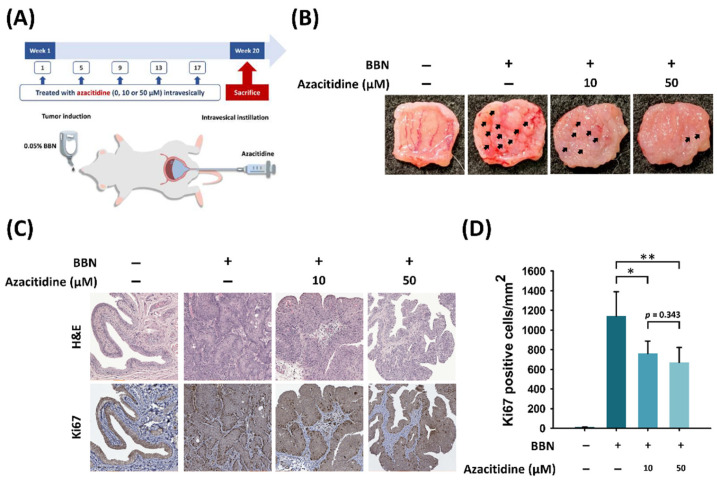
Azacitidine inhibits in vivo tumor growth and lesions in a rat BBN model of bladder cancer. (**A**) Flow chart of the animal model. Bladder tumors were induced in rats by supplying 0.05% BBN in their drinking water. Rats then underwent intravesical instillation of azacitidine (10 or 50 μM) at 1, 5, 9, 13, and 17 weeks and were sacrificed at week 20. (**B**) Images of bladder tumor lesions in each group. Azacitidine decreased the tumor lesions in the rat bladder. (**C**) Azacitidine instillation alleviated cell proliferation in the BBN-induced rat model, as indicated by H&E staining and immunohistochemistry staining for Ki67. (**D**) Azacitidine treatment alleviated the proliferation of bladder tumors. Quantitative analysis of Ki67-positive cells of bladder section in each group. Data are the mean ± s.d. (*p < 0.05; ** *p* < 0.01).

A preclinical test was developed to clarify the therapeutic potential of intravesical instillation (IVI) of azacitidine for the treatment of bladder cancer. The timeline and design of the experimental animal model are shown in Figure 5A. The rats were administered 0.05% BBN in their drinking water for 20 weeks to induce bladder cancer and were then given azacitidine by IVI once a month. Tumor lesions in the bladder were decreased by azacitidine treatment (Figure 5B). As shown in Figure 5C,D, immunohistochemistry staining using Ki67 as a marker to detect cell proliferation revealed that azacitidine significantly reduced the numbers of Ki67-positive cells compared to the control group when administered at 10 μM (1141 ± 249 vs. 760 ± 128 cells per mm^2^, *p* = 0.023) or 50 μM (1141 ± 249 vs. 669 ± 154 cells per mm^2^, *p* = 0.007) (Figure 5C,D).

## 4. Discussion

This study provides the first evidence that the IVI of azacitidine suppresses tumor formation through TNF-R1 and TRAIL R2 signaling in genotoxic carcinogen-induced bladder cancer. UBUC is an obstinate malignancy with a high recurrence rate under current intravesical therapy protocols and it has a poor prognosis. The findings presented here confirm the antiproliferative and apoptosis-activating activities of the DNMT1 inhibitor, azacitidine, in UBUC cell lines and implicated an involvement of the TNFR-NF-κB and TRAIL pathways in the underlying mechanism. Extension of these in vitro observations to an in vivo rat model confirmed that IVI of azacitidine resulted in a statistically significant reduction in the tumor lesions of the bladder and of the proliferation index marker Ki67. Taken together, our preliminary results validate the potential use of azacitidine as an agent for intravesical therapy of UBUC.

Azacitidine has shown growth-suppressing effects in several tumor types. Kratzsch et al., showed an antiproliferative effect of azacitidine in an in vitro experiment and in a xenograft animal model of glioblastoma [24]. Azacitidine also inhibited the proliferation of diffuse large B-cell lymphoma and germ cell tumor cell lines [25,26]. Our work has further extended the growth inhibitory effects of azacitidine to include UBUC (Figure 1). Azacitidine might inhibit proliferation through apoptosis or autophagy or by its cytotoxic effects due to incorporation into RNA [27]. Our findings suggest that azacitidine inhibited cell proliferation by inducing apoptosis (Figure 2).

Several studies have indicated that azacitidine drives apoptosis by various pathways. For example, an interferon-induced apoptotic pathway was implicated in epithelial cancers (e.g., ovarian, colorectal, and breast cancers) [28]. Another in vitro study revealed that azacitidine treatment may promote apoptosis in acute myeloid leukemia cells through caspase activation following azacitidine incorporation into RNA [29]. In hepatic cancer cells, azacitidine-induced apoptosis may be associated with expression of the Bcl-2 family proteins [30]. On the Fas-associated death domain, pro-caspase 8 is converted into caspase 8, which is responsible for cell apoptosis via the activation of caspase 3 [31]. Our work now provides another possibility by showing that azacitidine can promote apoptosis via the TRAIL pathway in UBUC (Figure 4C).

This apoptosis-related signaling pathway is initiated when TRAIL R2 assembles with its TRAIL ligand. Our work is supported by previous observations with detacibine (5-aza-2′-deoxycytidine), another DNMT1 inhibitor, which has shown a similar apoptosis-inducing ability via the TRAIL pathway in acute myeloid leukemia and in HRAS_G12V_-transformed cells [32,33]. Detacibine is a nucleoside analogue that has lost an oxygen atom at the position 2 carbon atom. In contrast to azacitidine, which incorporates primarily into RNA, detacibine incorporates predominantly into DNA [27].

In addition to the TRAIL pathway mechanism, our findings also implicate a downregulation of the NF-κB pathway by azacitidine in the UBUC cell lines (Figure 4A). This downregulation ultimately led to suppression of transcription factors p50 and p65 and downregulation of survivin, claspin, and HIF-1α (Figure 4B). Past research has clarified the role of survivin upregulation by the NF-kB pathway and suppression of apoptosis in bladder cancer [34]. In contrast to our findings, a canine bladder cancer investigation showed no changes in survivin expression following azacitidine treatment [35]. Claspin is an essential DNA-damage checkpoint protein that is degraded by the proteasome, and DNA damage is caused by apoptosis [36]. Previous work by Kenneth et al., showed a critical role for NF-κB modulation of the claspin mRNA level [37]. In their study, IKKβ reduction was associated with claspin downregulation.

Interestingly, in addition to azacitidine involvement in the pro-apoptosis pathway, other research has found that this drug might drive activation of the pro-survival pathway after long-term drug exposure in myelodysplastic syndrome patients [38]. The increased expression of pro-survival-associated proteins, including the mammalian target of rapamycin (mTOR), PLCγ, AKT, ERK, and transcription factor STAT3/5, was observed under long-term exposure of azacitidine [38]. These proteins are associated with proliferation and autophagy and might promote cancer cell survival. The cytotoxic activity of azacitidine was augmented when combined with an autophagy inhibitor in samples from myelodysplastic syndrome patients. The JAK-STAT pro-survival pathway is closely linked to cancer cell survival [39], and a recent study suggested that p-STAT, a downstream JAK-STAT pathway protein, bound to the promoter of DNMT1 and triggered tumor progression in glioblastoma [40]. Therefore, the pro-survival pathway might influence the promotor of DNMT1.

Another study has shown drug resistance to azacitidine in the treatment of myelodysplastic syndromes and acute myeloid leukemia [41]. Further investigation revealed that the autophagy inhibitor ROC-325 improved the anti-leukemic action of azacitidine in acute myeloid leukemia cells [42]. Interestingly, detacibine, an analogue of azacitidine, had a similar effect, as it induced autophagy of cancer cells, including UBUC cells [43,44]. The autophagy-inducing effect of detacibine was observed in mouse neuronal cell lines, which also showed increased hypoxia tolerance following detacibine treatment. Further investigation revealed that the increased hypoxia-tolerance-dependent autophagy involved the mammalian target of rapamycin (mTOR)-tuberous sclerosis complex 1 (TSC1) pathway.

In the present study, a BBN rat model was used to unravel the therapeutic role of IVI-administered azacitidine. The observed decrease in Ki67-positive cells demonstrated a possible in vivo inhibition of urothelial tumor proliferation by azacitidine. Previous work has shown a promising therapeutic effect of subcutaneous azacitidine in canine bladder carcinoma [45]. A major limitation of animal models is that a cross section of the bladder does not encompass the entire tumor; that is, some microlesions may be missed. The Ki67 index also has several defects, including different effects on the proliferation of different types of malignancies, and this index may be subjective [46]. Bladder cancer in humans has several risk factors, so one limitation of this model is that the BBN rat model does not mimic the tumorigenic condition of bladder tumors in humans, and oncogene expression in the rat model differs from that in human tumors [47].

IVI of azacitidine has advantages in clinical practice as it can avoid side effects, such as neutropenia and gastrointestinal symptoms, including nausea and vomiting. These were common adverse effects reported following oral administration of azacitidine in a clinical trial studying the therapeutic effect of oral azacitidine on acute myeloid leukemia in elderly patients [48]. Renal toxicity has also been reported, as both proximal and distal tubular damage have been observed in patients receiving systemic azacitidine [49]. In the present study, azacitidine was given by IVI and was expected to show fewer systemic effects than when administered by oral or intravenous routes.

A limitation of our in vitro study was that our cell lines consisted of specific types of UBUC; the T24 and UMUC3 cells are not fully representative of all UBUC types. Furthermore, the mechanism by which azacitidine affects the NF-κB or TRAIL pathways could not be determined without conducting methylation analysis of the entire UBUC genome. The molecular effects of azacitidine identified in vitro were not confirmed in our in vivo investigation, but this could be explained by the long duration (three weeks) between the last azacitidine exposure via IVI and sacrifice. We also did not investigate the potential azacitidine effects on the pro-apoptosis pathway and autophagy previously discussed in many references [48,49]. Thus, our inference of the possible pathway might be limited due to a lack of direct evidence.

## 5. Conclusions

Our study highlighted a possible mechanism of azacitidine effects on UBUC. Our work also provided novel therapeutic insights into the benefits of administration of azacitidine by IVI as a therapy for UBUC. Our findings may therefore expand the avenues of bladder cancer therapy.

## Data Availability

All data analyzed are included in this article and additional information is available upon request.
